# Correction to “4D Biofabrication of Magnetically Augmented Callus Assembloid Implants Enables Rapid Endochondral Ossification via Activation of Mechanosensitive Pathways”

**DOI:** 10.1002/advs.77066

**Published:** 2026-08-10

K. Ioannidis, A. Dimopoulos, I. Decoene, el al., 4D “Biofabrication of Magnetically Augmented Callus Assembloid Implants Enables Rapid Endochondral Ossification via Activation of Mechanosensitive Pathways,” *Adv* *anced* *Sci* *ence* 12, no. 15 (2025): 2413680, https://doi.org/10.1002/advs.202413680.

In the originally published article, pixel duplication was identified in the zoomed histological panel of the control spleen in Figure 8F. Following review of the original materials and clarification provided to the journal, the duplication was determined to have resulted from a microscope tile‐stitching artifact during mosaic image acquisition. To correct this issue and to ensure consistency across Figure 8F, the affected stitched/mosaic images were replaced with newly acquired single‐field images using identical imaging settings and post‐image processing conditions. Where no artifact or inconsistency was present, the corresponding image was retained. The corrected figure panel does not affect the interpretation of the data, the results, or the conclusions of the article. For transparency, both the originally published figure and the corrected figure are provided below.

Revised Figure panel 8F:


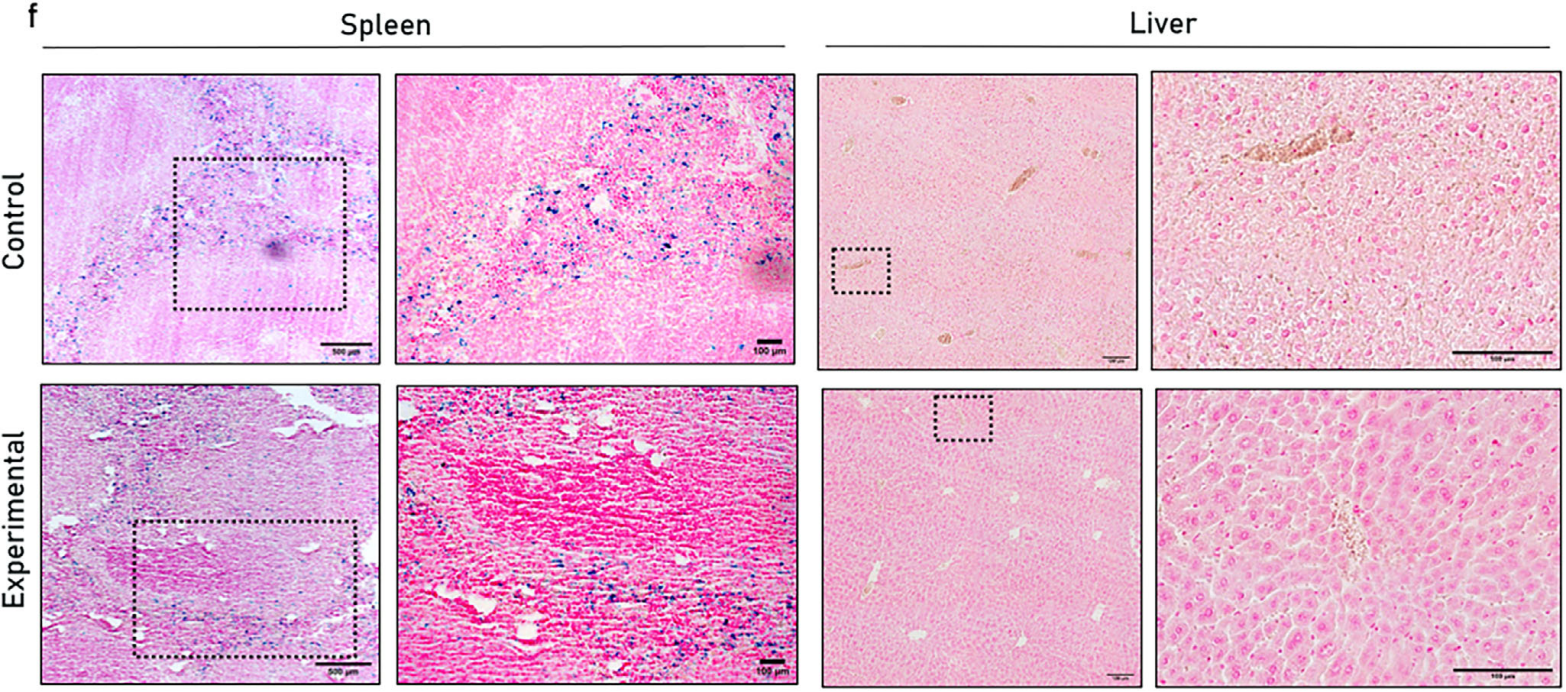


Original Figure panel 8F:


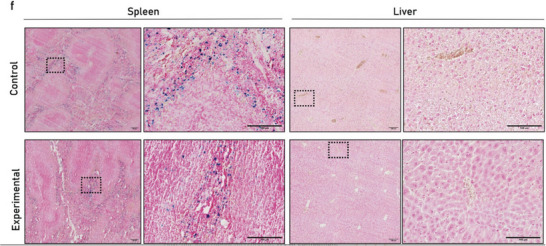


The authors apologize for this error and for any inconvenience it may have caused.

